# Refining quality control strategies in highly automated laboratories: experience in the integration of multistage statistical designs and risk management

**DOI:** 10.11613/BM.2025.030704

**Published:** 2025-10-15

**Authors:** María Costa-Pallaruelo, Álvaro García-Osuna, Marina Canyelles, Cecília Martínez-Bru, Nicoleta Nan, Rosa Ferrer-Perez, Francisco Blanco-Vaca, Leonor Guiñón

**Affiliations:** 1 Biochemistry Department, Hospital de la Santa Creu i Sant Pau, IIB Sant Pau, Barcelona, Spain; 2 Core Laboratory, Hospital de la Santa Creu i Sant Pau, Barcelona, Spain; 3 Quality Department Laboratories, Hospital de la Santa Creu i Sant Pau, Barcelona, Spain

**Keywords:** laboratory automation, Max E(Nuf) QC model, multistage QC, risk management, run size

## Abstract

**Introduction:**

The ISO 15189:2022 standard considers both the robustness of analytical methods and the risk of erroneous results in the quality control plan (QCP) design. Westgard *et al*.’s nomogram recommends quality control (QC) rules based on sample run size to ensure that the maximum expected number of unreliable patient results remains below one. This study aimed to implement a standardized, risk-based QC strategy across multiple analyzers without integrated on board QC, ensuring practical quality assurance.

**Material and methods:**

Thirty-two biochemistry parameters on Alinity c systems and three on Cobas Pro systems were included. The analytical performance of each parameter on each analyzer was assessed using sigma metric. Workload requirements were considered to determine the desired run size. Based on the “sigma metric statistical QC run size nomogram” proposed by Westgard *et al.*, a multistage bracketed QCP was designed for each parameter. When multiple designs were available, the simplest QC rule was prioritized.

**Results:**

Seven QCPs were initially established for 35 parameters. In the absence of automation, practical adaptations based on sigma metrics were implemented. Additionally, to streamline management, the QCP covering the greatest number of parameters *per* analyzer was prioritized, which ultimately resulted in the adoption of only two general QCP. Only 4 individualized QCP were required to cover 10 parameters with lower sigma values.

**Conclusions:**

This approach demonstrates the feasibility of implementing a refined QC strategy for parameters with sigma ≥ 4 in a highly automated laboratory, ensuring consistent quality assurance and efficient resource allocation for higher-risk tests.

## Graphical abstract

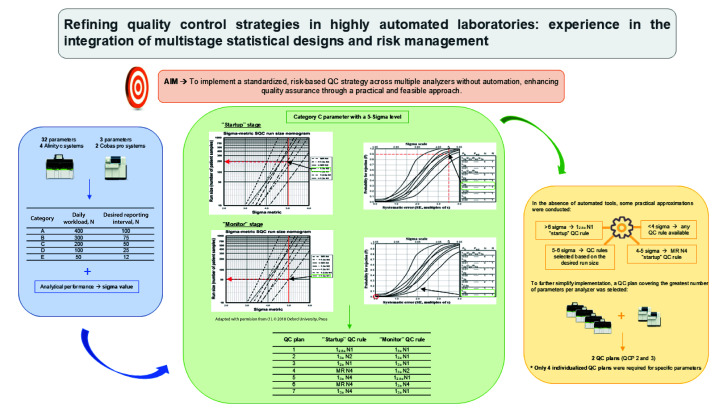

## Highlights

• We present a practical approach integrating multistage quality control (QC) design and run size requirements

• Two of the 7 QC plans initially designed were implemented across all analyzers

• Only 4 individualized QC plans were required for specific parameters

## Introduction

Highly automated laboratories operate in continuous mode, with uninterrupted data collection ensuring a steady flow of samples through analytical systems. This mode is characterized by real-time monitoring, where sample measurements are continuously performed, providing instant data. Continuous performance can be evaluated through a bracketed internal quality control (IQC), in which the results are not reported until the next scheduled IQC event is passed, ensuring that only reliable data are used for decision-making (*1*).

Proper quality control (QC) plans are essential to ensure reliable results for clinical decision-making, since these decisions have a direct impact on patient safety. However, despite the well-established principle that QC strategies should be tailored to analytical performance, many highly automated laboratories still apply a uniform QC rule and a fixed number of control measurements across all parameters, without accounting for the specific performance of each measurement procedure in each of the available analyzers (*1*-*4*). Notably, approximately 59% of laboratories worldwide continue to use the 1_2s_ rule for all measurands, without considering it to the performance characteristics of individual tests (*2*). Moreover, QC is usually managed with a one-stage approach, which means that all QC events are treated equally throughout the daily workload. Concerning this, Westgard *et al.* introduced the concept of multistage bracketed statistical QC combining a “startup” design for the initial phase of operation with a “monitor” design to maintain quality standards during the continuous reporting of patient test results (*1*). Starting the process is a critical control point where the probability of error is high so a more demanding QC design with a high probability of error detection (preferably > 90%) is applied. After the performance is assured by the “startup” QC and testing patient samples is initiated, then a less demanding “monitor” design with a lower probability of false rejection (< 5%) is applied.

Analytical performance is usually evaluated using the sigma metric; the higher the sigma, the better the performance, and *vice versa* (*3*, *5*). Nevertheless, based solely on analytical performance, the absence of errors during analysis cannot be guaranteed, so that erroneous patient results can be reported. In this regard, the ISO 15189:2022 standard (section 7.3.7.2.) requires considering not only the robustness and stability of the analytical method but also the risk of reporting erroneous results when designing QC plans (*6*). Additionally, the Clinical and Laboratory Standards Institute (CLSI) issued practice guidelines (C24-Ed4) to assist the implementation of risk management within QC plans (*7*).

In this regard, Parvin *et al.* introduced the concept of the number of unreliable final patient results (Nuf), referring to erroneous results that, once released, are unlikely to be identified and corrected. Building on this, the Max E(Nuf) value represents the maximum expected number of such unreliable results yielded between two QC events. A value of Max E(Nuf) below one indicates that, on average, fewer than one erroneous result is expected before error detection, thus ensuring acceptable analytical risk. Although it assumes normally distributed errors and consistent system performance - conditions that may not always hold in practice - this concept provides a standardized method for designing QC strategies, introducing sample run size as a key factor in QC plans (*8*, *9*). Defining an appropriate sample run size, the detection of errors is improved, thereby minimizing the reporting of erroneous patient results. To facilitate practical implementation, Westgard *et al.* developed a sigma-metric run size nomogram, which, based on the sample run size, recommends the appropriate QC rules, as well as the number of control measurements that are needed to maintain Max E(Nuf) below one (*1*).

This study is based on the hypothesis that a QC plan integrating multistage statistical designs and risk management can be effectively applied across multiple analyzers in the same laboratory, leading to a standardized and improved analytical control. The aim of this study was to integrate this optimized QC strategy in the absence of automated QC tools in a practical and feasible way in our laboratory, thus enhancing quality assurance.

## Materials and methods

### Materials

This study was conducted in the core laboratory of a tertiary hospital in Catalonia, Spain. This laboratory is dedicated to high-throughput automated testing and operates continuously - 24 hours a day, 365 days a year - processing both urgent and routine analyses. It is equipped with a track system that integrates the preanalytical, analytical, and postanalytical phases, and employs spectrophotometric and immunoassay platforms. Most tests (more than 75%) are performed within the biochemistry area, accounting for approximately 1000 orders daily.

Annually, the biochemistry area of the core laboratory performs more than 3,375,528 determinations (less than 0.5% from outpatients), covering a catalogue of over 177 tests. The facility is equipped with nine clinical chemistry analyzers and staffed by 11 specialists in biochemistry.

Internal quality control data were collected over a six-month period (September 2023 to March 2024). The IQC materials used included Multichem S Plus and Multichem U (Technopath Clinical Diagnostics, Ballina, Ireland), as well as PreciControl CARD, PreciControl PCT, and PreciControl TN (Roche Diagnostics GmbH, Mannheim, Germany). The same lot of IQC materials was used throughout the study, selected based on the availability of extensive historical performance data. Calibrators and reagents also remained consistent, supported by long shelf-life supplies.

### Methods

Twenty-eight biochemistry parameters in serum and seven in urine (U) were included in the study. Thirty-two parameters were measured in Alinity c systems (Abbott Laboratories, Chicago, USA). Three parameters were measured in Cobas Pro systems (Roche Diagnostics, Mannheim, Germany). Table 1 shows the distribution of the parameters based on the number of analyzers in which the measurement procedure was available.

**Table 1:** Distribution of measurement procedures across the analyzers in the laboratory

**One analyzer**	**Two analyzers**	**Three analyzers**	**Four analyzers**
Albumin U Calcium U Glucose U HDL Phosphorus U	Amylase Creatinine U Direct Bilirubin Potassium U NT-proBNP Procalcitonin Sodium U Troponin T Urea U Uric Acid	AST Chloride Cholesterol CK LD Magnesium Potassium Sodium Triglyceride	Albumin AP ALT Calcium Creatinine GGT Glucose CRP Total Bilirubin Total Protein Urea
HDL - high density lipoprotein. AP - alkaline phosphatase. ALT - alanine aminotransferase. AST - aspartate aminotransferase. CK - creatine kinase. LD - lactate dehydrogenase. GGT - gamma-glutamyl transferase. CRP - C-reactive protein. NT-proBNP - N-terminal pro brain natriuretic peptide. U - urine.			

To select an appropriate strategy, the first step was to evaluate the analytical performance of the measurement procedures using sigma metric. Thus, for each parameter imprecision and analytical bias were calculated for each concentration of the quality control levels and for each analyzer. Imprecision was expressed as coefficient variation (CV). Analytical bias, expressed as systematic error (SE), was determined as SE (%) = ((Mean value - Target value) / Target value) x100. Allowable total error (TEa) was obtained from the updated quality specifications and was adjusted according to the analytical performance of each measurement procedure (*10*-*12*). The most demanding TEa that ensured good analytical performance (sigma ≥ 4) was selected (*13*, *14*). Whenever specifications based on biological variation were unavailable, state-of-the-art specifications were adopted (*15*). Allowable total error and the source of the analytical performance specifications selected are detailed in Supplementary Tables 1 and 2. For each quality control level on each analyzer, the sigma value was calculated using the following equation: Sigma (σ) = (TEa - SE) / CV (*1*). Next, the mean sigma value for each analyzer was estimated, provided that no measurement procedure had a sigma value below 4 on any analyzer. As an exception, it was accepted that at low concentrations the sigma value could be between 3 and 4, since analytical performance is usually poorer in these concentration ranges and, for the parameters of the study, there are not concentration-dependent quality specifications that allow obtaining a more reliable sigma.

Once the analytical performance of each parameter was calculated, the workload requirements were considered to establish the appropriate run size for each measurement procedure. First, we established five categories considering different daily workloads and their corresponding sample run size, understood as the number of patient samples between two QC events, according to the model proposed by Westgard *et al.* (*1*). To simplify the quality control strategy, the sample run size was defined as a quarter of the daily workload, ensuring manageable monitoring. Based on the workload data, each parameter was assigned to one of the following run size categories (maximum of samples is in the brackets): A (100), B (75), C (50), D (25), and E (12). Thereafter, for each parameter a multistage QC plan was designed on the basis of the “sigma metric statistical QC run size nomogram” proposed by Westgard *et al.* (*1*).

The “startup” stage was designed considering a number of patient samples greater than or equal to the daily workload. Additionally, the selected QC rule should have had a probability of error detection (Ped) ≥ 0.9 to ensure the detection of the majority of analytical errors.

The “monitor” stage was designed considering a number of patient samples greater than or equal to the sample run size. In this case, the QC rule selected should have had a probability of false rejection (Pfr) ≤ 0.05, to ensure that quality remained acceptable by regularly reporting results.

When multiple designs were available for a parameter, priority was given to the simplest rule, even if it resulted in an increase in the number of control measurements, as long as this increase was manageable.

## Results

**Figure 1:**
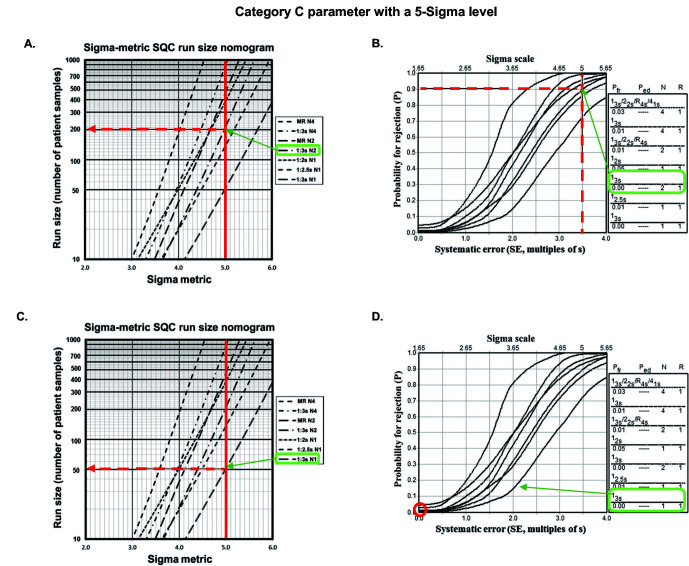
Application of Westgard nomogram: example for a category C parameter with 5-Sigma level. A and B illustrate the “startup” phase: (A) Intersection of workload (200 samples, Category C) and sigma determines the QC rule. (B) The power function graph confirms that this rule, at a sigma level of 5, achieves Ped ≥ 90%. C and D illustrate the “monitor” phase: (C) Intersection of sample run size (50 samples, Category C) and sigma determines the QC rule. (D) The power function graph confirms that this rule, at a sigma level of 5, achieves Pfr ≤ 5%. Adapted with permission from (*1*). Ped - probability of error detection. Pfr - probability of false rejection. QC - quality control. SE - systematic error. σ - sigma.

The mean sigma values obtained for each parameter in each of the analyzers are shown in Tables 2 and 3, respectively. All Alinity systems showed 6-10 parameters with sigma values ≥ 6, 5-9 parameters with sigma between 5 and 6, 1-5 parameters with sigma between 4 and 5 and up to 3 parameters with sigma < 4. Both Cobas Pro systems showed 1-2 tests reaching sigma values ≥ 6 and 1-2 parameters with sigma between 4 and 5. Based on these performance metrics and the workload category requirements, tailored QC plans combining “startup” and “monitor” QC rules were developed, as shown in Table 4, along with an explanation of the selected QC rules. Figure 1 illustrates how these QC plans were selected, using as an example of Category C parameter with a sigma value of 5. It outlines the development of appropriate QC rules for both the “startup” and “monitor” phases using the Westgard nomogram, and their validation through power function graphs to ensure compliance with predefined Ped and Pfr thresholds. Tables 5 and 6 illustrate the QC plan obtained for each parameter and analyzer, along with their appropriate workload management.

**Table 2:** Mean sigma values for the parameters measured on Alinity c systems

**Parameter**	**σ, mean**			
	**Alinity c1**	**Alinity c2**	**Alinity c3**	**Alinity c4**
Albumin	≥ 6	≥ 6	5.74	≥ 6
AP	≥ 6	5.26	≥ 6	5.66
ALT	≥ 6	≥ 6	≥ 6	≥ 6
Amylase	na	5.39	5.90	na
AST	≥ 6	na	4.38	5.52
Calcium	≥ 6	5.82	5.14	5.82
Cholesterol	na	5.24	4.92	5.65
Chloride	4.14	3.31	4.63	na
CK	na	≥ 6	≥ 6	≥ 6
Creatinine	≥ 6	5.00	5.42	5.45
Direct Bilirubin	5.15	na	5.03	na
GGT	≥ 6	4.19	≥ 6	≥ 6
Glucose	5.20	≥ 6	≥ 6	≥ 6
HDL	na	na	na	4.43
LD	3.69	na	3.93	5.58
Magnesium	3.39	na	4.36	4.68
CRP	5.00	5.83	5.27	5.86
Potassium	5.94	5.91	≥ 6	na
Sodium	3.16	3.66	3.48	na
Total Bilirubin	≥ 6	≥ 6	≥ 6	≥ 6
Total Protein	5.89	4.05	3.74	5.39
Triglyceride	≥6	na	5.59	4.51
Urea	≥ 6	≥ 6	5.11	5.94
Uric Acid	na	5.18	4.47	na
Albumin U	na	≥ 6	na	na
Calcium U	na	≥ 6	na	na
Creatinine U	≥ 6	≥ 6	na	na
Glucose U	na	≥ 6	na	na
Phosphorus U	na	5.23	na	na
Potassium U	≥ 6	≥ 6	na	na
Sodium U	≥ 6	5.55	na	na
Urea U	5.68	5.63	na	na
σ - sigma. AP - alkaline phosphatase. ALT - alanine aminotransferase. AST - aspartate aminotransferase. CK - creatine kinase. HDL - high density lipoprotein. LD - lactate dehydrogenase. GGT - gamma-glutamyl transferase. CRP - C-reactive protein. U - urine. na - parameter not available.				

**Table 3:** Mean sigma values for the parameters measured in Cobas Pro systems

**Parameter**	**σ, mean**	
	**Cobas 1**	**Cobas 2**
NT-proBNP	4.97	4.21
Procalcitonin	≥ 6	4.91
Troponin T	≥ 6	≥ 6
σ - sigma. NT-proBNP - N-terminal pro brain natriuretic peptide.		

**Table 4:** Quality control plans developed according to the “sigma metric statistical QC run size nomogram”

**QC plan**	**“Startup” QC rule**	**“Monitor” QC rule**
1	1_2.5s_ N1	1_3s_ N1
2	1_3s_ N2	1_3s_ N1
3	1_2s_ N1	1_3s_ N1
4	MR N4	1_3s_ N2
5	1_3s_ N4	1_2.5s_ N1
6	MR N4	1_3s_ N4
7	1_3s_ N4	1_3s_ N1
QC - quality control. N - number of quality control measurements. MR N4 represents a 1_3s_/2_2s_/R_4s_/4_1s_ multirule with 4 control measurements *per* QC event. 1_2.5s_ N1 is a 1_2.5s_ single rule with 1 control *per* QC event. 1_3s_ N2 is a 1_3s_ single rule with 2 control measurements *per* QC event. 1_2s_ N1 is a 1_2s_ single rule with 1 control *per* QC event; 1_3s_ N4 is a 1_3s_ single-rule procedure with 4 control measurements *per* QC event. 1_3s_ N1 is a 1_3s_ single rule with 1 control measurement *per* QC event.		

**Table 5:** Quality control plans for the parameters measured in Alinity c systems

**Parameter**	**Alinity c1**		**Alinity c2**		**Alinity c3**		**Alinity c4**	
	**QCP**	**Category**	**QCP**	**Category**	**QCP**	**Category**	**QCP**	**Category**
Albumin	1	B	1	C	1	C	1	D
AP	1	B	1	C	1	C	1	D
ALT	1	B	1	B	1	C	1	D
Amylase	na	na	1	D	1	E	na	na
AST	1	C	na	na	4	C	1	D
Calcium	1	B	1	C	3	C	1	D
Cholesterol	na	na	1	C	3	E	1	D
Chloride	4	E	-	E	7	E	na	na
CK	na	na	1	D	1	E	1	E
Creatinine	1	A	5	B	1	C	1	C
D Bil	2	E	na	na	3	E	na	na
GGT	1	B	6	C	1	C	1	D
Glucose	1	B	1	B	1	C	1	D
HDL	na	na	na	na	na	na	4	D
LD	-	C	na	na	-	E	1	E
Magnesium	-	C	na	na	4	E	7	E
CRP	3	C	1	C	2	C	1	D
Potassium	1	B	1	B	1	A	na	na
Sodium	-	B	-	B	-	A	na	na
T Bil	1	B	1	C	1	C	1	D
Total Protein	1	C	6	C	-	C	2	D
Triglyceride	1	C	na	na	1	E	4	D
Urea	1	A	1	A	3	C	1	C
Uric Acid	na	na	2	E	7	E	na	na
Albumin U	na	na	1	D	na	na	na	na
Calcium U	na	na	1	E	na	na	na	na
Creatinine U	1	E	1	E	na	na	na	na
Glucose U	na	na	1	E	na	na	na	na
Phos U	na	na	1	E	na	na	na	na
Potassium U	1	E	1	E	na	na	na	na
Sodium U	1	E	1	E	na	na	na	na
Urea U	1	E	1	E	na	na	na	na
AP - alkaline phosphatase. ALT - alanine aminotransferase. AST - aspartate aminotransferase. CK - creatine kinase. D Bil - direct bilirubin. HDL - high density lipoprotein. LD - lactate dehydrogenase. GGT - gamma-glutamyl transferase. CRP - C reactive protein. Phos – phosphorus. QCP - quality control plan. T Bil - total bilirubin. U - urine. na - not available. “-“ - sigma < 4.								

**Table 6:** Quality control plans for the parameters measured in Cobas Pro systems

**Parameter**	**Cobas 1**		**Cobas 2**	
	**QCP**	**Category**	**QCP**	**Category**
NT-proBNP	3	D	4	D
Procalcitonin	1	E	3	E
Troponin T	1	D	1	D
QCP - quality control plan. NT-proBNP - N-terminal pro brain natriuretic peptide.				

To demonstrate the feasibility of implementing QC plans integrating multistage designs and risk-based criteria across multiple analyzers, several practical adjustments were necessary in our setting due to the absence of automated tools. In the “startup” phase, although the 1_3s_ N1 rule could have been applied to parameters with sigma values ≥ 6 and daily workloads below 350, a unified approach was preferred (*16*). Therefore, the 1_2.5s_ N1 rule was selected for all such parameters, balancing simplicity and performance. During the “monitor” phase, the selection of QC rules was primarily based on the sample run size, followed by evaluation of the Pfr. The 1_2s_ N1 rule was excluded from this stage, given its relatively high Pfr (about 0.05), which may compromise routine efficiency. In both phases, when multiple rule options were available, the simplest rule achieving Ped ≥ 0.9 was consistently prioritized. These decisions, summarized in Table 7, illustrate how harmonized, analyzer-spanning QC strategies can be constructed in a practical and scalable manner while maintaining analytical rigor. To further simplify implementation across the six analyzers, and in line with the proposed harmonized QC strategy, the QC plan covering the greatest number of parameters *per* analyzer was selected. This resulted in the adoption of only two QC plans (QCP 2 and 3) out of the seven initially defined. As shown in Table 8, only ten parameters with lower sigma values required individualized QC plans due to their need for stricter QC rules. These ten parameters were effectively covered by four individual QCPs.

**Table 7:** Framework for selecting a quality control plan based on the sigma level of measurement procedures

**Sigma level**	**“Startup” stage**	**“Monitor” stage**
≥ 6	1_2.5s_ N1	QC rules could be selected based on the desired run size*
5-6	QC rules could be selected based on the desired run size*	QC rules could be selected based on the desired run size*
4-5	MR N4	QC rules could be selected based on the desired run size*
< 4	na^†^	na^†^
*QC rule could be selected based on the “sigma metric statistical QC run size nomogram” proposed by Westgard *et al* (1). ^†^No QC strategy is available, as no “startup” QC rule achieves a Ped ≥ 0.9. Improvement of the analytical performance is needed. QC - quality control. N - number of quality control measurements *per* QC event.		

**Table 8:** Quality control plan implemented by analyzer and individualized quality control plans for specific parameters

**Analyzer**	**QCP**	**Individualized QCP, parameter**
Alinity1 c1	3	4, chloride
Alinity1 c2	2	5, creatinine; 6, GGT and total protein
Alinity2 c3	3	4, AST and magnesium; 7, chloride and uric acid
Alinity3 c4	2	7, magnesium; 4, triglyceride and HDL
Cobas Pro1	3	-
Cobas Pro2	3	4, NT-proBNP
AST - aspartate aminotransferase. HDL - high density lipoprotein. NT-proBNP - N-terminal pro brain natriuretic peptide. QCP - quality control plan.		

## Discussion

Multistage bracketed statistical QC strategies, which integrate analytical performance metrics and workload requirements, offer a promising approach to optimize patient safety in clinical laboratories. This strategy allowed us to tailor QC plans, to achieve a balance between quality assurance and operational efficiency.

Several general concepts helped us implement the new QC strategies. First, to control a measurement procedure with a sigma value ≥ 6, simple QC rules with Ped ≥ 0.9 could be employed, resulting in Max E(Nuf) values from 0.3-0.4, which means that 250-333 patient samples could be analyzed between two QC events, ensuring that no more than one erroneous result would be reported (*16*). Second, for measurement procedures with sigma values between 5 and 6, QC rules can be selected based on the desired run size, while maintaining an acceptable Max E(Nuf). For measurement procedures with sigma values between 4 and 5, achieving an acceptable Max E(Nuf) requires applying more complex QC rules, such as multiple control rules 1_3s_/2_2s_/R_4s_/4_1s_ (MR N4), in the “startup” stage to reach an appropriate Ped, regardless of the desired run size. Finally, for sigma values below 4, no “startup” QC rule achieves a Ped ≥ 0.9, indicating poor performance and the need for improvements to ensure the quality of these measurement procedures to achieve a Max E(Nuf) <1 (*1*, *17*). These principles formed the basis for the selection and standardization of QC plans across the analyzers.

Some of the advantages of such a QC strategy are related to its scalability and long-term efficiency. While the initial implementation effort increases with the number of analytes and analytical platforms, once established, the addition of new parameters or analyzers requires considerably less effort. This is because key components of the strategy, such as the QC plan categories derived from a risk-based classification model, along with the QC rule selection criteria, are already standardized and can be systematically applied across different systems (*1*). Moreover, free QC calculators available on the Westgard website offer a user-friendly tool to facilitate the application of risk-based QC rule selection and performance evaluation (*18*). Although these calculators were not used in our study, they may help other laboratories in integrating risk-based QC strategies. Additionally, by allocating more demanding QC plans only to parameters with lower sigma values, the strategy ensures optimal use of resources without compromising analytical quality (*1*, *19*).

Despite these advantages, several limitations must be acknowledged. The lack of automated tools constituted the major limitation for the implementation of this QC strategy in our laboratory. Automated measurement of on-board controls based on the category assigned at each parameter and analyzer, along with the management of rules for accepting or rejecting control results, would enable us an individualized management of each parameter (*20*). Performing these functions manually requires specialized laboratory personnel and entails considerable effort. In our setting, we addressed this in Alinity systems by configuring the middleware AlinIQ AMS (Abbott Laboratories, Chicago, USA) to withhold sample results once the predefined number of measurements *per* category was performed for each parameter and analyzer. These rules enabled us to lock the corresponding test until the required QC plan was completed, ensuring compliance with established QC plan. Furthermore, as our hospital does not provide primary care services, sample flow remains generally consistent, with predictable peak times throughout the day, facilitating the timely execution of QC procedures. For the Cobas Pro systems, where no middleware is available, this procedure was performed manually by laboratory staff according to the same predefined rules.

While the lack of automation was a primary constraint, additional factors may impact the broader applicability of our findings. Although the sigma metric remains a useful tool for QC design, its reliability depends on the veracity of imprecision and bias estimates, that changes constantly, as well as, on the appropriate selection of the TEa. These variables can significantly affect sigma values and, consequently, influence the selection of QC plan (*19*, *21*).

Other authors have demonstrated the practical applicability of a multistage bracketed statistical QC model in their laboratories. Nevertheless, these studies were predominantly focused on the optimization of the QC plan for an isolated parameter or for a set of parameters within a single analyzer (*22*, *23*). To date, no study has been published showing the implementation of this QC strategy across multiple parameters and the several analytical systems of a highly automated laboratory.

Future work should focus on the development or adaptation of middleware tools that support dynamic QC plan assignment based on real-time performance metrics. Moreover, integrating economic evaluations, such as cost-effectiveness or cost-benefit analyses, into the model could strengthen its practical value and support decision-making in resource-limited settings. Finally, the implementation of this approach in similar laboratories with diverse analytical platforms and operational context would provide valuable information to confirm the model’s applicability and guide further refinement.

This pragmatic approach demonstrates the feasibility of implementing a refined QC strategy in a highly automated laboratory for all parameters with a sigma level ≥ 4. It offers a practical framework for optimizing laboratory performance by ensuring consistent quality assurance and enabling focused allocation of resources to parameters with higher analytical risk. Further validation across diverse laboratory settings will help confirm its broader applicability and inform future enhancements.

## Data Availability

The datasets generated during the current study are available from the corresponding author on reasonable request.
