# Corrigendum to: Refining quality control strategies in highly automated laboratories: experience in the integration of multistage statistical designs and risk management

**DOI:** 10.11613/BM.2026.021201

**Published:** 2026-04-15

**Authors:** Maria Costa-Pallaruelo, Alvaro Garcia-Osuna, Marina Canyelles, Cecilia Martinez-Bru, Nicoleta Nan, Rosa Ferrer-Perez, Francisco Blanco-Vaca, Leonor Guinon

**Affiliations:** 1 Biochemistry Department, Hospital de la Santa Creu i Sant Pau, IIB Sant Pau, Barcelona, Spain; 2 Core Laboratory, Hospital de la Santa Creu i Sant Pau, Barcelona, Spain; 3 Quality Department Laboratories, Hospital de la Santa Creu i Sant Pau, Barcelona, Spain

This is a correction of Biochem Med (Zagreb) 2025;35(3):030704. DOI: https://doi.org/10.11613/BM.2025.030704.

Since the publication of the article, the authors have noticed an error in the calculation of the sigma metric. The corrected calculations are presented below. We apologize to the readers for any inconvenience this error may have caused.

During a post-publication review of the calculations, an error was identified in the formula used for the calculation of the sigma metric. Specifically, the calculations were performed without considering the absolute value in the formula: Sigma (σ) = (TEa − |SE|) / CV (where TEa represents the allowable total error, SE the systematic error and CV the coefficient of variation).

Consequently, the sigma values reported for the evaluated parameters were affected. After identifying this issue, the sigma metrics were recalculated using the correct formula including the absolute value of SE. Subsequently, all related analyses and procedures based on these sigma values were repeated using the corrected calculations.

All corrections are underlined to facilitate identification of the changes from the previously published version.

## Corrections in the Abstract

Results: Additionally, to streamline management, the QCP covering the greatest number of parameters per analyzer was prioritized, which ultimately resulted in the adoption of only three general QCP. Only 4 individualized QCP were required to cover 9 parameters with lower sigma values.

## Corrections in the Methods section

For each quality control level in each analyzer, the sigma value was calculated using the equation: Sigma (σ) = (TEa - |SE|) / CV.

## Corrections in the Results section

The mean sigma values obtained for each parameter in each of the analyzers are shown in Tables 2 and 3, respectively. All Alinity systems showed 9 to 14 parameters with sigma values ≥ 6, 4 to 8 parameters with sigma between 5 and 6, 3 to 5 parameters with sigma between 4 and 5 and up to 2 parameters with sigma < 4.

This resulted in the adoption of only three QC plans (QCP 1, 2 and 3) out of the seven initially defined. As shown in Table 8, only nine parameters with lower sigma values required individualized QC plans due to their need for stricter QC rules. These nine parameters were effectively covered by four individual QCPs.

## Corrections in the Discussion section

Second, for measurement procedures with sigma values between 4.65 and 6, QC rules can be selected based on the desired run size, while maintaining an acceptable Max E(Nuf). For measurement procedures with sigma values between 4 and 4.65, achieving an acceptable Max E(Nuf) requires applying more complex QC rules, such as multiple control rules 13s/22s/R4s/41s (MR N4), in the “startup” stage to reach an appropriate Ped, regardless of the desired run size.

## Corrections in Tables

**Table 2:** Mean sigma values for the parameters measured in Alinity c systems

**Parameter**	**σ, mean**			
	**Alinity c1**	**Alinity c2**	**Alinity c3**	**Alinity c4**
Albumin	≥ 6	≥ 6	5.56	5.39
AP	≥ 6	5.30	≥ 6	5.94
ALT	4.75	4.68	5.81	5.16
Amylase	na	≥ 6	≥ 6	na
AST	≥ 6	na	4.97	≥ 6
Calcium	5.84	5.66	5.14	5.82
Cholesterol	na	5.06	4.51	4.96
Chloride	4.14	3.31	4.63	na
CK	na	≥ 6	≥ 6	≥ 6
Creatinine	≥ 6	≥ 6	≥ 6	≥ 6
Direct Bilirubin	≥ 6	na	≥ 6	na
GGT	≥ 6	4.19	≥ 6	≥ 6
Glucose	5.61	≥ 6	≥ 6	≥ 6
HDL	na	na	na	4.21
LD	≥ 6	na	≥ 6	≥ 6
Magnesium	3.00	na	2.45	3.31
CRP	≥ 6	≥ 6	≥ 6	≥ 6
Potassium	5.77	5.81	5.32	na
Sodium	3.12	3.49	3.22	na
Total Bilirubin	≥ 6	≥ 6	≥ 6	5.14
Total Protein	≥ 6	≥ 6	≥ 6	≥ 6
Triglyceride	≥ 6	na	≥ 6	≥ 6
Urea	≥ 6	≥ 6	4.78	5.69
Uric Acid	na	≥ 6	4.55	na
Albumin U	na	5.54	na	na
Calcium U	na	≥ 6	na	na
Creatinine U	≥ 6	≥ 6	na	na
Glucose U	na	≥ 6	na	na
Phosphorus U	na	5.23	na	na
Potassium U	≥ 6	≥ 6	na	na
Sodium U	4.61	5.00	na	na
Urea U	5.62	5.21	na	na
σ - sigma. AP - alkaline phosphatase. ALT - alanine aminotransferase. AST - aspartate aminotransferase. CK - creatine kinase. HDL - high density lipoprotein. LD - lactate dehydrogenase. GGT - gamma-glutamyl transferase. CRP - C-reactive protein. U - urine. na - parameter not available.				

**Table 5:** Quality control plans for the parameters measured in Alinity c systems

**Parameter**	**Alinity c1**		**Alinity c2**		**Alinity c3**		**Alinity c4**	
	**QCP**	**Category**	**QCP**	**Category**	**QCP**	**Category**	**QCP**	**Category**
Albumin	1	B	1	C	1	C	1	D
AP	1	B	1	C	1	C	1	D
ALT	5	B	5	B	1	C	2	D
Amylase	na	na	1	D	1	E	na	na
AST	1	C	na	na	3	C	1	D
Calcium	1	B	1	C	2	C	1	D
Cholesterol	na	na	2	C	4	E	2	D
Chloride	4	E	-	E	7	E	na	na
CK	na	na	1	D	1	E	1	E
Creatinine	1	A	1	B	1	C	1	C
D Bil	1	E	na	na	1	E	na	na
GGT	1	B	6	C	1	C	1	D
Glucose	1	B	1	B	1	C	1	D
HDL	na	na	na	na	na	na	4	D
LD	1	C	na	na	1	E	1	E
Magnesium	-	C	na	na	-	E	-	E
CRP	1	C	1	C	1	C	1	D
Potassium	1	B	1	B	2	A	na	na
Sodium	-	B	-	B	-	A	na	na
T Bil	1	B	1	C	1	C	2	D
Total Protein	1	C	1	C	1	C	1	D
Triglyceride	1	C	na	na	1	E	1	D
Urea	1	A	1	A	5	C	1	C
Uric Acid	na	na	1	E	4	E	na	na
Albumin U	na	na	1	D	na	na	na	na
Calcium U	na	na	1	E	na	na	na	na
Creatinine U	1	E	1	E	na	na	na	na
Glucose U	na	na	1	E	na	na	na	na
Phos U	na	na	1	E	na	na	na	na
Potassium U	1	E	1	E	na	na	na	na
Sodium U	7	E	2	E	na	na	na	na
Urea U	1	E	1	E	na	na	na	na
AP - alkaline phosphatase. ALT - alanine aminotransferase. AST - aspartate aminotransferase. CK - creatine kinase. D Bil – direct bilirubin. HDL - high density lipoprotein. LD - lactate dehydrogenase. GGT - gamma-glutamyl transferase. CRP - C reactive protein. Phos – phosphorus. QCP - quality control plan. T Bil - total bilirubin. U - urine. na - not available. “-“ - sigma < 4.								

**Table 7:** Framework for selecting a quality control plan based on the sigma level of measurement procedures

**Sigma level**	**“Startup” stage**	**“Monitor” stage**
≥ 6	1_2.5s_ N1	QC rules could be selected based on the desired run size*
4.65-6	QC rules could be selected based on the workload*	QC rules could be selected based on the desired run size*
4-4.65	MR N4	QC rules could be selected based on the desired run size*
< 4	na^†^	na^†^
*QC rule could be selected based on the “sigma metric statistical QC run size nomogram” proposed by Westgard et al (1). †No QC strategy is available, as no “startup” QC rule achieves a Ped ≥ 0.9. Improvement of the analytical performance is needed. QC - quality control. N - number of quality control measurements per QC event.		

**Table 8:** Quality control plan implemented by analyzer and individualized quality control plans for specific parameters

**Analyzer**	**QCP**	**Individualized QCP, parameter**
Alinity1 c1	1	5, ALT; 4, chloride; 7, sodium U
Alinity1 c2	2	5, ALT; 6, GGT
Alinity2 c3	3	4, cholesterol and uric acid; 5, urea; 7, chloride
Alinity3 c4	2	4, HDL
Cobas Pro1	3	-
Cobas Pro2	3	4, NT-proBNP
ALT - alanine aminotransferase. HDL - high density lipoprotein. NT-proBNP - N-terminal pro brain natriuretic peptide. QCP - quality control plan.		

**Supplementary table 1:** Allowable total error (and source of the analytical performance specification) applied to each parameter measured in Alinity c systems

	**TEa**	**Source**
Albumin	8.25	p90 SEQC
AP	21.00	MCS
ALT	18.70	EFLM des
Amylase	13.10	EFLM des
AST	18.70	EFLM min
Calcium	9.10	MCS
Cholesterol	8.30	EFLM des
Chloride	6.00	MCS
CK	8.50	p90 SEQC
Creatinine	22.00	MCS
D Bil	23.40	EFLM des
GGT	9.20	EFLM opt
Glucose	9.30	EFLM min
HDL	14.90	EFLM min
LD	21.00	MCS
Magnesium	9.40	MCS
CRP	26.00	EFLM opt
Potassium	7.30	EFLM min
Sodium	3.20	MCS
T Bil	37.00	EFLM min
Total Protein	9.00	MCS
Triglyceride	26.20	EFLM des
Urea	17.10	EFLM des
Uric Acid	12.50	EFLM des
Albumin U	14.00	MCS
Calcium U	16.00	MCS
Creatinine U	13.00	MCS
Glucose U	8.40	MCS
Phos U	11.00	MCS
Potassium U	8.60	MCS
Sodium U	7.20	MCS
Urea U	13.00	MCS
AP - alkaline phosphatase. ALT - alanine aminotransferase. AST - aspartate aminotransferase. CK - creatine kinase. CRP - C-reactive protein. D Bil - direct bilirubin. EFLM des - desirable analytical performance specification (APS) according to the European Federation of Clinical Chemistry and Laboratory Medicine (EFLM) biological variation database (10). EFLM min - minimum APS according to the EFLM biological variation database (10). EFLM opt - optimal APS according to the EFLM biological variation database (10). HDL - high density lipoprotein. LD - lactate dehydrogenase. GGT - gamma-glutamyl transferase. MCS - Spanish minimum consensus specification, based on state-of-the-art performance (12). p90 SEQC - 90th percentile of measurement errors provided by the Spanish Society of Laboratory Medicine (SEQCML) from the external quality assessment schemes, used as a state-of-the-art APS. Phos – phosphorus. QCP - quality control plan. SOTA - state-of-the-art. T Bil - total bilirubin. TEa - allowable total error. U – urine.		

